# Biogenesis of diverse plant phasiRNAs involves an miRNA-trigger and Dicer-processing

**DOI:** 10.1007/s10265-016-0878-0

**Published:** 2016-11-29

**Authors:** Reina Komiya

**Affiliations:** Science and Technology Group, Okinawa Institute of Science and Technology Graduate University (OIST), 1919-1 Tancha, Kunigami-gun, Okinawa 904-0495 Japan

**Keywords:** phasiRNAs, tasiRNAs, 22-nt miRNAs, DCL-processing, ARGONAUTE, Reproduction

## Abstract

It has been almost 30 years since RNA interference (RNAi) was shown to silence genes via double-stranded RNAs (dsRNAs) in *Caenorhabditis elegans* (Fire et al. 1998). 20–30-nucleotide (nt) small non-coding RNAs are a key element of the RNAi machinery. Recently, phased small interfering RNAs (phasiRNAs), small RNAs that are generated from a long RNA precursor at intervals of 21 to 26-nt, have been identified in plants and animals. In *Drosophila*, phasiRNAs are generated by the endonuclease, Zucchini (Zuc), in germlines. These phasiRNAs, known as one of PIWI-interacting RNAs (piRNAs), mainly repress transposable elements. Similarly, reproduction-specific phasiRNAs have been identified in the family Poaceae, although DICER LIKE (DCL) protein-dependent phasiRNA biogenesis in rice is distinct from piRNA biogenesis in animals. In plants, phasiRNA biogenesis is initiated when 22-nt microRNAs (miRNAs) cleave single-stranded target RNAs. Subsequently, RNA-dependent RNA polymerase (RDR) forms dsRNAs from the cleaved RNAs, and dsRNAs are further processed by DCLs into 21 to 24-nt phasiRNAs. Finally, the phasiRNAs are loaded to ARGONAUTE (AGO) proteins to induce RNA-silencing. There are diverse types of phasiRNA precursors and the miRNAs that trigger the biogenesis. Their expression patterns also differ among plant species, suggesting that species-specific combinations of these triggers dictate the spatio-temporal pattern of phasiRNA biogenesis during development, or in response to environmental stimuli.

## Introduction

In the early 1990s, RNA silencing was reported as co-suppression and quelling in plants and fungi. Transgenes result in the suppression of endogenous transcripts with homologous transgene sequences (Napoli et al. 1990; Romano and Macino 1992). In *Caenorhabditis elegans*, double-stranded RNAs (dsRNAs) induce RNA silencing, called RNA interference (RNAi) (Fire et al. 1998). Small RNAs are key players in the RNAi machinery, and play pivotal roles during various developmental stages and in pathogenesis. In the plant RNAi pathway, 21–24-nucleotide (nt) small RNAs are produced via processing of dsRNAs by DICER LIKE (DCL) proteins encoding RNaseIII. Processed small RNAs are loaded onto Argonaute proteins (AGOs) and small RNA-AGO complexes trigger RNA-silencing through RNA cleavage or DNA methylation in a small RNA sequence-dependent manner (Castel and Martienssen 2013; Czech and Hannon 2011).

RNA silencing is classified into two types: transcriptional gene silencing (TGS) and post-transcriptional gene silencing (PTGS). In *Arabidopsis thaliana,* 24-nt repeat-associated siRNAs (rasiRNAs) bound to AGO4 cause RNA-directed DNA methylation (RdDM) and TGS. TGS, mediated by rasiRNAs via DNA methylation, suppresses many transposable elements (TEs) and repeat regions to prevent their transposition and transmission to the next generation (Matzke et al. 2009). In contrast, PTGS silences genes via target-RNA cleavage and/or translational repression (Iwakawa and Tomari 2015). In PTGS induced by exogenous factors, such as transgenes or viral infections, dsRNAs formed in template exogenous RNAs by RNA-dependent RNA polymerase (RDR) and DCL-dependent processing result in production of 21-nt, small interfering RNAs (siRNAs) for defense against viral and exogenous transgenes (Hamilton and Baulcombe 1999). PTGS is also triggered by 21- or 22-nt micro RNAs (miRNAs), derived from hairpin structures, or 21-nt *trans*-acting siRNAs (tasiRNAs), which are one type of phased small interfering RNAs (phasiRNAs) in plants.

**Fig. 1:**
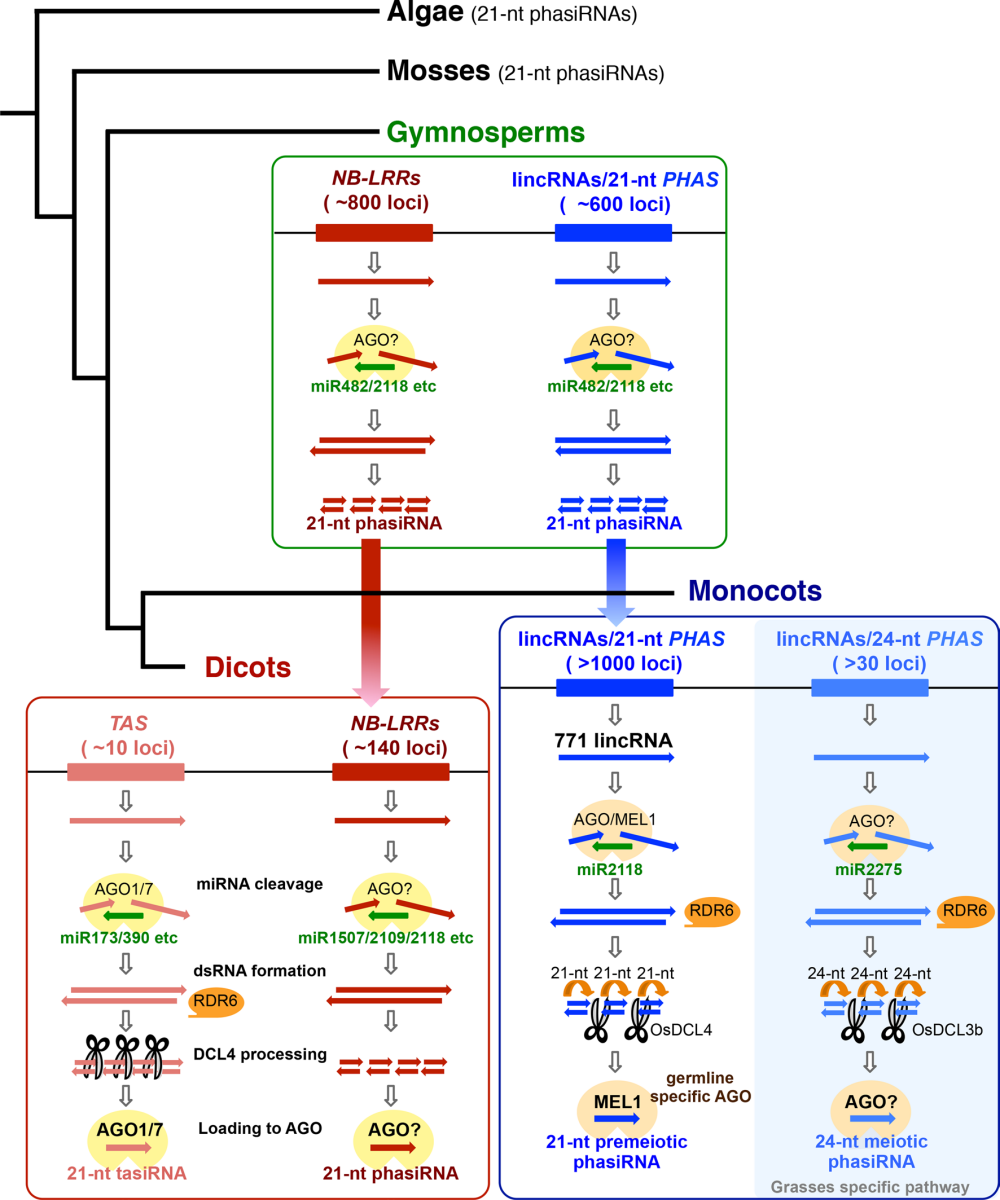
A model for phasi/tasiRNA biogenesis in gymnosperms, dicots, and monocots. In plant phasi/tasiRNA biogenesis, (1) phasiRNA precursor transcripts are cleaved by 22-nt miRNA, (2) (RDR)-dependent dsRNAs are synthesized, (3) DCL-dependent processing results in phasiRNAs and (4) their loading onto AGO proteins. The phasiRNA pathway is conserved in gymnosperms, dicots, and monocots. In gymnosperms, there are miR482/miR2118-triggered phasiRNAs derived from both coding-RNAs (NB-LRRs, etc.) and non-coding RNAs (reproductive lincRNAs). The phasiRNA pathway, involving miR482/miR2118-triggered coding-RNAs (NB-LRRs), is also conserved in dicots. In contrast, miR482/miR2118-triggered phasiRNAs generated from reproductive lincRNAs are conserved in monocots

phasiRNAs are endogenous, eukaryote small RNAs that occur at intervals of 21–26 nucleotides. tasiRNAs, which are well-studied in *Arabidopsis thaliana*, play a role in leaf phase transition from juvenile to adult phases of vegetative stages (Peragine et al. 2004). In *Arabidopsis thaliana* tasiRNA biogenesis, single-stranded, non-coding RNAs are transcribed from *TAS* intergenic loci and cleaved by an AGO1/7-miRNA complex. RDR6 recruits cleaved *TAS* transcripts and forms dsRNAs. dsRNA formation follows DCL4-dependent processing into tasiRNAs 21-nt in length. Subsequently AGO1/7 associated with 21-nt tasiRNAs causes PTGS by cleaving target RNAs, such as transcripts of auxin response factor (ARF) family, pentatricopeptide repeat (PPR) genes, and MYB transcription factors that include complement sequences recognized by AGO1/7-tasiRNAs complexes (Allen and Howell 2010; Allen et al. 2005; Vazquez et al. 2004; Yoshikawa et al. 2005), (Fig. 1). Recently, many types of phasiRNAs that are arranged at regular 21-, 22-, or 24-nt phased intervals have been identified in various plant species (International Brachypodium Initiative 2010; Johnson et al. 2009; Xia et al. 2015; Zhai et al. 2011; Zheng et al. 2015). Plant phasiRNA biogenesis generally involves 22-nt miRNA cleavage of a phasiRNA precursor RNA, dsRNA synthesis by RDR6, and processing by DCLs. This review focuses on the biogenesis and diversity of phasiRNAs in plants and animals.

### 21-nt and 24-nt reproductive phasiRNAs in Poaceae

Numerous phasiRNAs are produced specifically during reproductive stages in the family Poaceae (International Brachypodium Initiative 2010; Johnson et al. 2009; Komiya et al. 2014; Song et al. 2012a, b; Ta et al. 2016; Zhai et al. 2015; Zheng et al. 2015). Johnson et al. (2009) reported that 21- and 24-nt phasiRNAs, which are specifically expressed in rice panicles, are derived from over 1000 intergenic regions, collectively termed *PHAS* loci. Most of the large intergenic non-coding RNAs (lincRNAs, *PHAS*) identified as precursor RNAs are single-stranded. They are transcribed with poly(A) tails without introns during early reproductive stages prior to meiosis in rice (Komiya et al. 2014). The precursor lincRNAs include 22-nt miR2118 recognition motifs, and the 24-nt phasiRNAs precursors also contain 22-nt miR2275 recognition motifs, where miR2118/miR2275 cleaves the precursor RNA to trigger the biogenesis of 21-nt/24-nt phasiRNAs. Subsequently, after dsRNA formation of cleaved RNAs, OsDCL4 generates 21-nt phasiRNAs or OsDCL3b produces 24-nt phasiRNAs (Johnson et al. 2009; Song et al. 2012a, b). DCLs-dependent phasiRNAs often form small RNA-duplexes with 2-bp overhangs at both 3′-termini. It is an intriguing subject how DCL4 and DCL3b recognize the differences of the dsRNAs derived from 21-nt *PHAS* or 24-nt *PHAS* in future.

Angiosperms develop primordial germ cells within reproductive organs that differentiate into meiocytes. Subsequently, meiocytes generate haploid gametophytes or spores via meiosis. MEIOSIS ARRESTED AT LEPTOTENE1 (MEL1) is a rice AGO protein that functions in the development of pre-meiotic germ cells and the progression of meiosis in both male and female organs. MEL1 binds dominantly to 21-nt phasiRNAs bearing a 5′-terminal cytosine, although AGOs interacting with 24-nt phasiRNAs remain unknown (Komiya et al. 2014; Nonomura et al. 2007), (Fig. 1). phasiRNA biogenesis in rice, such as 22-nt miRNA cleavage, OsDCL processing and AGO loading, is very similar to tasiRNA pathway in *Arabidopsis thaliana*. In rice, expression of miR2118, lincRNAs and MEL1 coincides with premeiotic stages when germ cells differentiate, while this expression is very low in other stages. On the other hand, OsDCL4 regulates rice leaf development, spikelet formation, and stamen development in both vegetative and reproductive stages (Komiya et al. 2014; Liu et al. 2007; Nagasaki et al. 2007; Song et al. 2012a, b; Toriba et al. 2010). These results suggest that miR2118/lincRNAs/MEL1 regulates reproductive-specificities of 21-nt phasiRNA biogenesis, although functions of 21- and 24-nt phasiRNAs during premeiotic and meiotic stages in monocot reproduction are not well understood.

Photoperiod-sensitive male sterility (PSMS) is a phenomenon where particular rice strains show male sterility under long-day conditions, but are fertile under short-day conditions. Long-day-specific male-fertility-associated RNA (*LDMAR*) encodes the 1.2-kbp non-coding RNA that regulates PSMS. The abundant *LDMAR* transcript induces male sterility during long days, and is processed into small RNAs (Ding et al. 2012; Zhou et al. 2012). Interestingly, the phenotype of vacuolation of sporophytic germ-cells in *mel1* mutants is also observed in pollen mother cells (PMC) of *pms3* mutants, although the male sterility phenotype in *pms3* is distinct from that of *mel1* in both male and female tissues. (Ding et al. 2012; Nonomura et al. 2007). Further study might reveal whether the function of reproductive specific lincRNAs generating phasiRNAs is related to *LDMAR* that produces small RNAs in PSMS.

### Small RNAs interact with AGOs that function during plant reproduction

Ten AGO genes are present in the *Arabidopsis thaliana* genome, 19 in rice, and 17 in maize (Fei et al. 2013; Kapoor et al. 2008). *Arabidopsis thaliana* AGO5 (AtAGO5), related to rice MEL1, is believed to function in female gametogenesis, since a semi-dominant *ago5*-*4* mutant is defective in the initiation of mega-gametogenesis (Borges et al. 2011; Tucker et al. 2012). AtAGO2 is involved in somatic DNA repair, and also in chiasma frequency in PMC (Oliver et al. 2014; Wei et al. 2012). AtAGO9, which interacts with 24-nt rasiRNAs, restricts the number of spore mother cells in somatic cells of an ovule, suppresses the activity of transposable elements, and is also responsible for the frequency of chromosome interlocks in meiosis in PMC (Oliver et al. 2014; Olmedo-Monfil et al. 2010). Maize AGO104, which is likely orthologous to AtAGO9 and AtAGO4, accumulates in somatic tissues surrounding female meiocytes and regulates condensation and disjunction of meiotic chromosomes (Singh et al. 2011), (Table 1). Thus, some plant AGOs have specific functions during reproduction, including germ cell development, meiosis, and gametogenesis. No PIWI-related proteins have been identified in plant genomes.

**Table 1:** Small RNAs and AGOs in germlines of animals and plants

Small RNA	Size (nt)	Processing	AGO	AGO function	Species
Phased primary piRNAs	~26	Zucchini	PIWI, Aub	TE repression	*Drosophila*
piRNAs	21	ND	PRG-1	Non-self RNAs including transgene repression	*C. elegance*
rasiRNAs	24	ND (DCL3?)	AGO9	Germ cell fate repression and TE repression	*A. thaliana*
1C-small RNAs	21	ND (DCL4?)	AGO5	Female gametogenesis regulation	*A. thaliana*
phasiRNAs	21	OsDCL4	MEL1	Germ cell development and progression in meiosis	Rice
phasiRNAs	24	OsDCL3b/OsDCL5	ND	ND	Rice
ND (rasiRNAs?)	ND	ND	AGO104	Female germ cell development and chromosome condensation during meiosis	Maize
Premeiotic phasiRNAs	21	ND	ND (AGO5c?)	ND	Maize
Meiotic phasiRNAs	24	ND	ND (AGO18b?)	ND	Maize

*ND* no data

In *Arabidopsis thaliana*, AGO1/7-tasiRNAs *trans*-regulate target RNAs via cleavage. MEL1-phasiRNA is localized mainly to the cytoplasm in PMC, so the leading hypothesis is that phasiRNAs may cause target-RNA cleavages in *trans* with MEL1 in the cytoplasm. However, a parallel analysis of RNA ends (PARE) degradome, a modified RACE that detects cleaved transcripts, and a transcriptome analysis of *mel1* and *dcl4* mutants, have failed so far to identify any cleaved target transcripts with complementary sequences of these phasiRNAs, indicating that MEL1-phasiRNAs may not *trans*-regulate targets (Komiya et al. 2014; Song et al. 2012a).

Although MEL1 is mainly localized in the cytoplasm of PMC, it is transiently localized in the nuclei of meiocytes at leptotene stage. In *mel1* mutants, homologous chromosome synapsis and chromosome reprogramming via H3K9 (histone H3 lysine 9) modifications are depleted in early meiosis I (Komiya et al. 2014; Liu and Nonomura 2016). At the nearly same stage (zygotene) in maize meiocytes, DNA methylation increased at *PHAS* loci (Dukowic-Schulze et al. 2016). These results suggest that phasiRNAs regulate meiotic chromosome structure through epigenetic modifications to promote normal meiosis, although lincRNA expression was unaffected in panicles of *mel1* mutants. The functions of 21- and 24-nt phasiRNAs, including MEL1-phasiRNAs in plants is an important subject for future studies in order to understand a molecular mechanism of monocots reproduction and a role of intergenic regions.

### Phased piRNAs in animal germlines

piRNAs are germline-specific 23-30-nt small RNAs found in animals. piRNAs interacting with PIWI proteins repress expression of transposons to preserve genome integrity. Primary piRNAs are generated from single-stranded precursor transcripts derived from genomic transposons, and secondary piRNAs are amplified by the ping-pong pathway via PIWI proteins, Aubergene (Aub) and AGO3 in *Drosophila* germlines (Ghildiyal and Zamore 2009; Ishizu et al. 2012; Malone and Hannon 2009; Sato et al. 2015; Siomi et al. 2011; Thomson and Lin 2009). Recently, it was reported that phased piRNA generation is initiated by secondary piRNA-guided transcript cleavage, where every ~26-nt phaised piRNA are produced in the Zucchini endonuclease-dependent mechanism in *Drosophila* (Han et al. 2015; Mohn et al. 2015), (Table 1). During phased piRNA production, diverse piRNA sequences are produced, which allows quick adaptation to changes in transposon sequences. In *C. elegans*, Piwi-family AGO PRG-1 interacts with 21-nt piRNAs having uracil in the first position, and triggers RDR-dependent 22-nt RNA production having guanine in the first position, which suppresses transgenes (Gu et al. 2009; Ruby et al. 2006). Zucchini-dependent production of phased piRNAs indicates that phased piRNA biogenesis in animals is distinct from reproduction-specific phasiRNA biogenesis via miRNA-cleavage and DCLs processing in plants (Table 1).

### Evolution and diversification of phasiRNAs in plants

In *Medicago truncatula* (Fabaceae), the 22-nt miR1507, miR2109, and miR2118 act as triggers to initiate generation of 21- and 22-nt phasiRNAs from mRNAs that encode nucleotide binding and leucine-rich repeat (NB-LRR) pathogen-defense genes and these 22-nt miRNAs are conserved in other legumes and nonlegumes, members of the family Solanaceae (Fei et al. 2015; Zhai et al. 2011). Besides NB-LRR genes, MYB transcription factors, PPR genes and a number of single- or low-copy genes, including Ca2 + ATPase, have been identified as coding RNA precursors that produce phasiRNAs in dicots (Arikit et al. 2014; Fei et al. 2013; Xia et al. 2012; Zhu et al. 2012). These findings indicate that phasiRNAs are produced from protein-coding genes in dicots, in contrast to the Poaceae where non-coding RNAs are the main phasiRNA precursors (*Brachypodium*, rice and maize). The functions of phasiRNAs derived from the large gene families of MYB and PPR remain unknown. In contrast, NB-LRR gene families are regulated by both triggered-miRNAs and phasiRNAs generated from NB-LRRs, and the phasiRNAs also regulates in *trans* at other NB-LRR loci in legumes. Thus, NB-LRR-phasiRNA regulation shows a self-reinforcing network involving *cis*- or *trans*- regulation (Fei et al. 2013; Zhai et al. 2011).

A recent study using miR1507, miR2109 and miR2118 over expression lines and miRNA-targets, NB-LRRs, pairing analysis by validating the miRNA-precursor interaction, showed that miRNA levels limit the abundance of phasiRNAs (Fei et al. 2015). These results suggest that 22-nt miRNA abundance and/or efficiency of cleaving precursors RNAs are also key regulators for phasiRNA biogenesis.

In Norway spruce (*Picea abies*), over 2000 phasiRNA-produced loci (*PHAS* loci) have been identified, including ~800 loci derived from NB-LRRs and ~600 from non-coding RNAs, recognized by 41 species of miRNAs. Interestingly, miR418/miR2118 super-families in gymnosperms target both NB-LRR RNAs and reproductive non-coding RNAs, resulting in production of phasiRNAs derived from both NB-LRR RNAs and reproductive non-coding RNAs (Xia et al. 2015), (Fig. 1). These results suggest that dual miR482/miR2118-triggered phasiRNA biogenesis in gymnosperms emerged more than 300 million years ago, and that the pathways utilizing reproductive non-coding RNAs in monocots and the NB-LRR family-phasiRNA pathway in dicots may have been retained after the angiosperm lineage diverged (Fig. 1).

21-nt phasiRNAs are expressed in a wide range of plants, including algae, mosses, gymnosperms, basal angiosperms, monocots, and dicots. AGOs, DCLs, and RDRs required for phasiRNA biogenesis are also conserved from mosses to higher plants (Zheng et al. 2015), indicating that phasiRNA biogenesis is an ancient pathway in plants. In contrast, 24-nt phasiRNAs only exist in grass species (Arikit et al. 2013; Johnson et al. 2009; Zheng et al. 2015). Furthermore, miR2275 and DCL3b, involved in 24-nt meiotic phasiRNA production, are absent in dicot genomes, suggesting that the 24-nt phasiRNAs pathway originated recently in grasses or other monocots.

In maize anthers, two classes of phasiRNAs are expressed: 21-nt “premeiotic” phasiRNAs and 24-nt “meiotic” phasiRNAs, which are derived from a few hundred intergenic loci. LincRNAs (21-nt *PHAS*) and 21-nt phasiRNAs accumulate in pollen sacs, and miR2118 localizes in epidermis of premeiotic anthers in maize and African rice. In contrast, lincRNAs (24-nt *PHAS*), miR2275, and 24-nt phasiRNAs are expressed in tapetum and meiocytes in meiotic anthers (Ta et al. 2016; Zhai et al. 2015). Expression profiles of maize genes show that maize AGO5c, related to rice MEL1, is expressed at premeiotic stages and the expression timing and sites of AGO18b are similar to 24-nt meiotic phasiRNAs, suggesting that these AGOs may be candidates for binding to 21-nt premeiotic phasiRNAs/24-nt meiotic phasiRNAs (Table 1). Intriguingly, the two types of grass phasiRNAs are analogous to the mammalian piRNA classes, i.e. premeiotic 26 to 27-nt piRNAs and meiotic 29~30-nt piRNAs (Zhai et al. 2014, 2015). These two classes of small RNAs involved in male development may have resulted from convergent evolution of the reproductive small RNA pathway, which shares developmental timing and special expression patterns in male reproductive tissues in both plant and animal lineages. During land plant evolution, spatio-temporal regulation of phasiRNA biogenesis might have been helpful for plants to adapt to environmental changes and various stresses. Further studies to elucidate biogenesis and function of phasiRNAs would help us to understand their contribution to plant development and defence responses.

## Concluding remarks

Transcriptomic analyses of small RNAs have revealed that numerous kinds of phasiRNAs are expressed in plants. phasiRNA biogenesis, including 22-nt miRNA-cleavage, RDR-dsRNA synthesis, and DCL-processing, is conserved in gymnosperms, monocots, and dicots, suggesting its essential role in plants. In many cases, however, molecular mechanisms of action and phasiRNA functions remain unclear. Open questions include: (1) How are numerous lincRNAs regulated during specific developmental stages and pathogenesis? (2) What is the molecular mechanism of lincRNA cleavage by 22-nt miRNAs? (3) What is the primary role of phasiRNAs in plants? Answering these questions will require experiments exploiting technologies such as genomics/epigenetics using non-coding RNA mutants, cell sorting, biochemistry, and structural analysis. Such studies are expected to help us to understand the significance of non-coding regions in both plants and animals.
